# Umbilical cord‐derived MSC and hyperbaric oxygen therapy effectively protected the brain in rat after acute intracerebral haemorrhage

**DOI:** 10.1111/jcmm.16577

**Published:** 2021-05-02

**Authors:** Hon‐Kan Yip, Kun‐Chen Lin, Pei‐Hsun Sung, John Y. Chiang, Tsung‐Cheng Yin, Re‐Wen Wu, Kuan‐Hung Chen

**Affiliations:** ^1^ Division of Cardiology Department of Internal Medicine Kaohsiung Chang Gung Memorial Hospital and Chang Gung University College of Medicine Kaohsiung Taiwan; ^2^ Institute for Translational Research in Biomedicine Kaohsiung Chang Gung Memorial Hospital Kaohsiung Taiwan; ^3^ Center for Shockwave Medicine and Tissue Engineering Kaohsiung Chang Gung Memorial Hospital Kaohsiung Taiwan; ^4^ Department of Medical Research China Medical University Hospital China Medical University Taichung Taiwan; ^5^ Department of Nursing Asia University Taichung Taiwan; ^6^ Division of Cardiology Department of Internal Medicine Xiamen Chang Gung Hospital Xiamen China; ^7^ Department of Anesthesiology Kaohsiung Chang Gung Memorial Hospital and Chang Gung University College of Medicine Kaohsiung Taiwan; ^8^ Department of Computer Science and Engineering National Sun Yat‐Sen University Kaohsiung Taiwan; ^9^ Department of Healthcare Administration and Medical Informatics Kaohsiung Medical University Kaohsiung Taiwan; ^10^ Department of Orthopaedic Surgery Kaohsiung Chang Gung Memorial Hospital and Chang Gung University College of Medicine Kaohsiung Taiwan

**Keywords:** hyperbaric oxygen, intracerebral haemorrhage, mesenchymal stem cells, neurological function

## Abstract

This study tested the hypothesis that combined therapy with human umbilical cord‐derived mesenchymal stem cells (HUCDMSCs) and hyperbaric oxygen (HBO) was superior to either one on preserving neurological function and reducing brain haemorrhagic volume (BHV) in rat after acute intracerebral haemorrhage (ICH) induced by intracranial injection of collagenase. Adult male SD rats (n = 30) were equally divided into group 1 (sham‐operated control), group 2 (ICH), group 3 (ICH +HUCDMSCs/1.2 × 10^6^ cells/intravenous injection at 3h and days 1 and 2 after ICH), group 4 (ICH +HBO/at 3 hours and days 1 and 2 after ICH) and group 5 (ICH +HUCDMSCs‐HBO), and killed by day 28 after ICH. By day 1, the neurological function was significantly impaired in groups 2‐5 than in group 1 (*P* < .001), but it did not differ among groups 2 to 5. By days 7, 14 and 28, the integrity of neurological function was highest in group 1, lowest in group 2 and significantly progressively improved from groups 3 to 5 (all *P* < .001). By day 28, the BHV was lowest in group 1, highest in group 2 and significantly lower in group 5 than in groups 3/4 (all *P* < .0001). The protein expressions of inflammation (HMGB1/TLR‐2/TLR‐4/MyD88/TRAF6/p‐NF‐κB/IFN‐γ/IL‐1ß/TNF‐α), oxidative stress/autophagy (NOX‐1/NOX‐2/oxidized protein/ratio of LC3B‐II/LC3B‐I) and apoptosis (cleaved‐capspase3/PARP), and cellular expressions of inflammation (CD14+, F4/80+) in brain tissues exhibited an identical pattern, whereas cellular levels of angiogenesis (CD31+/vWF+/small‐vessel number) and number of neurons (NeuN+) exhibited an opposite pattern of BHV among the groups (all *P* < .0001). These results indicate that combined HUCDMSC‐HBO therapy offered better outcomes after rat ICH.

## 1 INTRODUCTION

Intracranial haemorrhage, one kind of stroke resulted from subarachnoid haemorrhage or intracerebral haemorrhage (ICH), is a highly unacceptable cause of death and disability in adults with an annual incidence of 10‐40 per 100, 000 population.^1^, ^2^, ^3^ In intracranial haemorrhage, the bleeding is usually derived from arterioles or small arteries directly into the brain, forming hematomas that spread along white matter pathway. The hematoma frequently continues to grow until the pressure surrounding it increases enough to limit its spread or until the haemorrhage decompresses itself by emptying into the ventricular system or into the cerebrospinal fluid.^2^, ^4^ Additionally, intracranial haemorrhage, always sabotages the brain tissue as it enlarges, that is first primary injury to the brain is the mechanical damage.^2^ The pressure created enough by blood and surrounding brain oedema is life‐threatening.^4^ The secondary injuries result from cytotoxicity of blood,^5^, ^6^ impaired calcium homeostasis,^7^ excitotoxicity from excitatory neurotransmitters (eg glutamate) ^8^, ^9^ and oxidative stress and inflammation.^6^, ^9^, ^10^, ^11^, ^12^, ^13^, ^14^ This neuroinflammation is up‐regulated, resulting in the release of cytokines, chemokines, cellular adhesion molecules (CAMs) and matrix metalloproteases (MMPs).^15^ Expression of MMPs increases the permeability of the BBB, allowing peripheral leucocytes to invade the area of injury, where they up‐regulate present inflammatory processes.^15^ Additionally, CAMs allow leucocytes to adhere to local vessels, permitting those cells to attract more cells to the site of injury.^15^ These aforementioned issues which are complicated could explain why the effective treatment of intracranial haemorrhage is still an unmet need.

Hypoxic neurons performing anaerobic metabolism result in acidosis, lactate production and an unsustainable reduction in cellular metabolic reserve.^16^ As the hypoxic microenvironment persists, the neuronal cells lose their ability to maintain ionic homeostasis, follow by free oxygen radical accumulation and cell membrane degradation,^16^ resulting in an irreversible cell death.^16^ This gives some basis to the assertion that therapy designed to increase oxygen availability in the early period following brain haemorrhage may improve long‐term outcomes.^17^ Interestingly, hyperbaric oxygen therapy (HBO) has been reported to be effective in improving the outcomes after traumatic brain injury.^17^ Another study has reviewed that HBO could be a new look on treating ischaemic stroke and traumatic brain injury.^18^ HBO is the therapeutic administration of 100% oxygen at environmental pressures greater than 1 atmosphere absolute (ATA) in an airtight vessel. In this way, it is possible to deliver a greatly increased partial pressure of oxygen to the tissue, including the ischaemic brain tissues.

Mesenchymal stem cell (MSC) has the capacity to attenuate inflammation^19^, ^20^, ^21^, ^22^, ^23^, ^24^ and down‐regulate innate and adaptive immunity^19^, ^21^, ^22^, ^23^, ^24^, ^25^, ^26^ through suppressing immunogenicity.^19^, ^21^, ^22^, ^23^, ^24^, ^25^, ^26^ Experimental studies have further demonstrated that MSCs therapy markedly preserved neurological function and reduced brain infarct volume in rodent after acute ischaemic stroke attack.^27^, ^28^ The results of these previous reports^19^, ^21^, ^22^, ^23^, ^24^, ^25^, ^26^, ^27^, ^28^ raise the hypothesis that MSCs therapy may offer unexpected benefit for patients after ICH, especially for those who have large ICH zone non‐candidate for surgical intervention. However, prior to apply MSC therapy for human being in a clinical setting of ICH, a preclinical study had to be first performed with human being‐derived MSC (ie xenogeneic MSC) to prove not only the safety and efficacy but also the immune privilege of the MSC regardless for what kind of biological species. Furthermore, as a result of the complexity of the pathophysiologic mechanisms involved in the haemorrhagic stroke, a single therapeutic strategy would be inadequate for disease improvement. Therefore, we performed a combined therapy with human umbilical cord blood‐derived stem cell (HUCDMSCs) and HBO for rodent after acute ICH.

## 2 MATERIALS AND METHODS

### 2.1 Ethics statement

All animal experimental procedures were approved by the Institutional Animal Care and Use Committee at Kaohsiung Chang Gung Memorial Hospital (Affidavit of Approval of Animal Use Protocol No. 2 018 032 102) and performed in accordance with the Guide for the Care and Use of Laboratory Animals, 8th edition (NIH publication No. 85‐23, National Academy Press, Washington, DC, USA, revised 2011). Animals were housed in an Association for Assessment and Accreditation of Laboratory Animal Care International‐approved animal facility in our hospital, with controlled temperature and light cycles (24°C and 12/12 light/dark cycle).

### 2.2 Procedure and protocol of animal model of intracerebral haemorrhage (ICH) induced by type IV collagenase proteolytic enzyme

The procedure and protocol of ICH were based on the previous report.^29^ In details, the rats were anaesthetized by inhalation of 2.0% isoflurane and then were placed on a warming pad at 37°C, followed by securing the head and shaving the scalp hair. Under sterile conditions, 1 cm long midline incision of the scalp was carefully created to expose the perpendicular intersection point of the coronal and sagittal suture (ie bregma). The Hamilton syringe (250 μL) was then mounted onto the injection pump and stereotaxically guided the needle (26 Gauge) over bregma. The stereotactic manipulator arms have adjusted the position of the needle 1.4 mm anterior and 3.2 mm lateral to the right. A small cranial burr hole was then created by using a 1 mm drill bit. After this procedure, 1.0 μL collagenase type IV (0.25 IU/μL) was then carefully injected into corpus/dorsal striatum (5 mm below the skull) by Hamilton syringe 26 G at a rate of 0.2 μL/min. The syringe was removed slowly after the injection is completed and sterile bone wax is used to plug the hole quickly. The skin on the surface of head was then closed by using 4‐0 prolene suture. Finally, the animals were cared for in a portable animal intensive care unit (ThermoCare**®**) with food and water for 24 hours.

### 2.3 Animal grouping

Pathogen‐free, adult male Sprague Dawley (SD) rats (n = 30) weighing 320‐350 g (Charles River Technology, BioLASCO Taiwan Co. Ltd., Taiwan) were used in the present study. The animals were equally categorized into group 1 [sham‐operated control (SC), ie only incision of the skin over scalp], group 2 [acute ICH induced by directly intracranial injection of collagenase (1.0 μL)], group 3 [ICH +HUCDMSCs (1.2 × 10^6^ cells) by intravenous administration at 3 hours and on days 1 and 2, respectively after ICH procedure, ie total amount was 3.6 × 10^6^ cells], group 4 **[**ICH +hyperbaric oxygen therapy (HBO) (3h duration for each time) at 3 h and at days 1 to 4 after ICH procedure] and group 5 (ICH +combined HUCDMSCs +HBO). Animals in each group were killed by day 28 after ICH induction and the brain specimen was harvested from each animal for individual study.

### 2.4 Hyperbaric oxygen therapy

The procedure and protocol of HBO therapy were based on a previous report.^30^ Briefly, to induce tissue‐level hyperoxia, SD rats were subjected to HBO administration in an animal tabletop chamber (Piersol‐Dive, model 4934) with the animals exposed to 100% oxygen at 2.4 atmospheres absolute (ATA) for 90 minutes (3 hour/one time) at 3 hours and days 1–4 after ICH induction.

### 2.5 Corner test for assessment of neurological function prior to and after ICH induction

The sensorimotor functional test (corner test) was conducted for each rat of each group (ie n = 6 per group) at baseline and on days 1, 7, 14 and 28 after acute ICH induction as we previously described.^28^, ^30^, ^31^ In detail, the rat could walk through a tunnel and then turn into a 60‐degree corner. To exit the corner, the rat could turn either left or right. The results were recorded by a technician blinded to the study design. This test was repeated 10‐15 times with at least 30 seconds between each trial. We recorded the number of right and left turns from 10 successful trials for each animal and used the results for statistical analysis.

### 2.6 Measurement of brain haemorrhagic area

To evaluate the impact of HUCDMSCs‐HBO treatment on preserving the brain parenchyma, coronal sections of the brain were obtained from four extra animals in each group as 2 mm slices by day 14 after ICH induction. Each cross section of brain tissue was then stained with 2% 3,5‐Triphenyl‐2H‐Tetrazolium Chloride (TTC) (Alfa Aesar) for brain haemorrhagic area (BHA) analysis. Briefly, all brain sections were placed on a tray with a scaled vertical bar to which a digital camera was attached. The sections were photographed from directly above at a fixed height. The images obtained were then be analysed using Image Tool 3 (IT3) image analysis software (University of Texas, Health Science Center, San Antonio, UTHSCSA; Image Tool for Windows, version 3.0, USA).

The haemorrhagic area was observed as either whitish or pale reddish regions (ie dis‐coloured region). Intracerebral haemorrhagic region was further confirmed by microscopic examination. The percentages of haemorrhagic area were then obtained by dividing the area with total cross‐sectional area of the brain. The rest of the brain tissue was then cut into pieces for specific studies.

### 2.7 Western blot analysis

The procedure and protocol for Western blot analysis were based on our recent reports.^28^, ^30^, ^31^, ^32^, ^33^ Briefly, equal amounts (50 μg) of protein extracts were loaded and separated by SDS‐PAGE using acrylamide gradients. After electrophoresis, the separated proteins were transferred electrophoretically to a polyvinylidene difluoride membrane (GE, UK). Non‐specific sites were blocked by incubation of the membrane in blocking buffer [5% non‐fat dry milk in T‐TBS (TBS containing 0.05% Tween 20)] overnight. The membranes were incubated with the indicated primary antibodies [Caspase 3 (1:1000, Cell Signaling), Poly (ADP‐ribose) polymerase (PARP) (1:1000, Cell Signaling), high mobility group box 1 (HMGB1) (1:1000, Cell Signaling), Toll‐like receptor (TLR)‐2 (1:1000, Abcam), TLR‐4 (1:1000, Abcam), myeloid differentiation primary response 88 (MyD88) (1:1000, Abcam), TNF receptor associated factor 6 (TRAF6) (1:1000, Abcam), IκB‐ß (1:1000, Abcam), nuclear factor (NF)‐κB (1:600, Abcam), interferon (INF)‐ γ (1:1000, Abcam), LC3B‐I (1:1000, Cell Signaling), LC3B‐II (1:1000, Cell Signaling), oxidized protein (1:200, Millipore), tumour necrosis factor (TNF)‐α (1:1000, Cell Signaling), interleukin (IL)‐1ß (1:1000, Cell Signaling), NOX‐1 (1:1500, Sigma), NOX‐2 (1:750, Sigma) and Actin (1:1000, Millipore)] for 1 hour at room temperature. Horseradish peroxidase‐conjugated anti‐rabbit immunoglobulin IgG (1:2000, Cell Signaling, Danvers, MA, USA) was used as a secondary antibody for one‐hour incubation at room temperature. The washing procedure was repeated eight times within one hour. Immunoreactive bands were visualized by enhanced chemiluminescence (ECL; Amersham Biosciences, Amersham, UK) and exposed to Biomax L film (Kodak, Rochester, NY, USA). For the purpose of quantification, ECL signals were digitized using Labwork software (UVP, Waltham, MA, USA).

### 2.8 Immunofluorescent (IF) staining of brain specimens

The procedure and protocol of IF staining were based on our previous reports.^28^, ^30^, ^31^, ^32^, ^33^ In detail, frozen sections (4 μm thick) was obtained from the brain haemorrhagic area/at‐risk area of each animal, permeated with 0.5% Triton X‐100, and incubated with antibodies against NueN (1:100, Merck), CD31 (1:100, Abcam), von Willebrand factor (vWF) (1:200, Abcam), CXCR4 (1:100, Invitrogen), CD14 (1:200, ProteinTech) and F4/80 (1:100, Santa Cruz) at 4ºC overnight. Alexa Fluor488, Alexa Fluor568 or Alexa Fluor594‐conjugated goat anti‐mouse or rabbit IgG was used to localize signals. Sections were finally counterstained with DAPI and observed with a fluorescent microscope equipped with epifluorescence (Olympus IX‐40). Three brain sections were analysed for each rat. For quantification, three randomly selected high‐power fields (HPFs; 400× for IF study) were analysed in each section. The mean number of positively stained cells per HPF for each animal was then be determined by summation of all numbers divided by 9.

### 2.9 Vessel density in brain infarct zone

The procedure and protocol for identifying number of small vessels in the brain haemorrhagic area/at‐risk area were based on our previous reports.^31^, ^32^, ^33^ In detail, staining of small blood vessels was performed with alpha smooth muscle actin (α‐SMA) (1:400) as primary antibody at room temperature for 1 hour, followed by washing with PBS thrice. Ten minutes after the addition of anti‐mouse‐HRP conjugated secondary antibody, the tissue sections were washed with PBS thrice. Then 3,3’ diaminobenzidine (DAB) (0.7 gm/tablet) (Sigma) will be added, followed by washing with PBS thrice after one minute. Finally, haematoxylin was added as a counter‐stain for nuclei, followed by washing twice with PBS after 1 min. Three brain sections were analysed in each rat. For quantification, three randomly selected HPFs (100×) were analysed in each section. The mean number per HPF for each animal was then be determined by summation of all numbers divided by 9.

### 2.10 Procedure and protocol of brain magnetic resonance imaging (MRI) for determining the brain haemorrhagic volume (BHV)

The procedure and protocol for brain magnetic resonance imaging (MRI) study were based on our previous report.^28^ The MRI was performed at day 28 after IS induction. Briefly, during MRI measurements, mice were anaesthetized by 2% inhalational isoflurane with room air and placed in an MRI‐compatible holder (Biospec 94/20, Bruker, Ettingen, Germany). Rectal temperature and respiration were monitored throughout the procedure to ensure normal physiological conditions were maintained. MRI data were collected using a Varian 9.4T animal scanner (BioSpec 94/20, Bruker, Ettingen, Germany) with a rat surface array. The MRI protocol consisted of 40 T2‐weighted images. Forty continuous slice locations were imaged with a field‐of‐view of 30 × 30 mm, an acquisition matrix dimension of 256 × 256 and slice thickness of 0.5 mm. The repetition time (TR) and echo time (TE) for each fast spin‐echo volume were 4200 and 30 ms, respectively. Custom software, ImageJ (1.43i, NIH, USA), was used to process the region of interest (ROI). Planimetric measurements of images from MRI T2 were performed to calculate the stroke volumes of cortex. Collectively, the BHV was calculated by summation of total coronal sections and then divided the numbers of coronal sections to obtain the means of infarct areas. Additionally, the height of the infarct zone was calculated by summation of the thickness of each coronal sections. Finally, the BHV was obtained by mean of infarct area x height.

### 2.11 Statistical analysis

Quantitative data were expressed as means ±SD. Statistical analysis was adequately performed by one‐way ANOVA, followed by Bonferroni multiple‐comparison post hoc test. SAS statistical software for Windows version 8.2 (SAS Institute, Cary, NC) was utilized. A *P* value of less than 0.05 was considered statistically significant.

## 3 RESULTS

### 3.1 Identification of haemorrhagic area by day 14 after ICH induction and time courses of neurological function assessed by corner test

**FIGURE 1:**
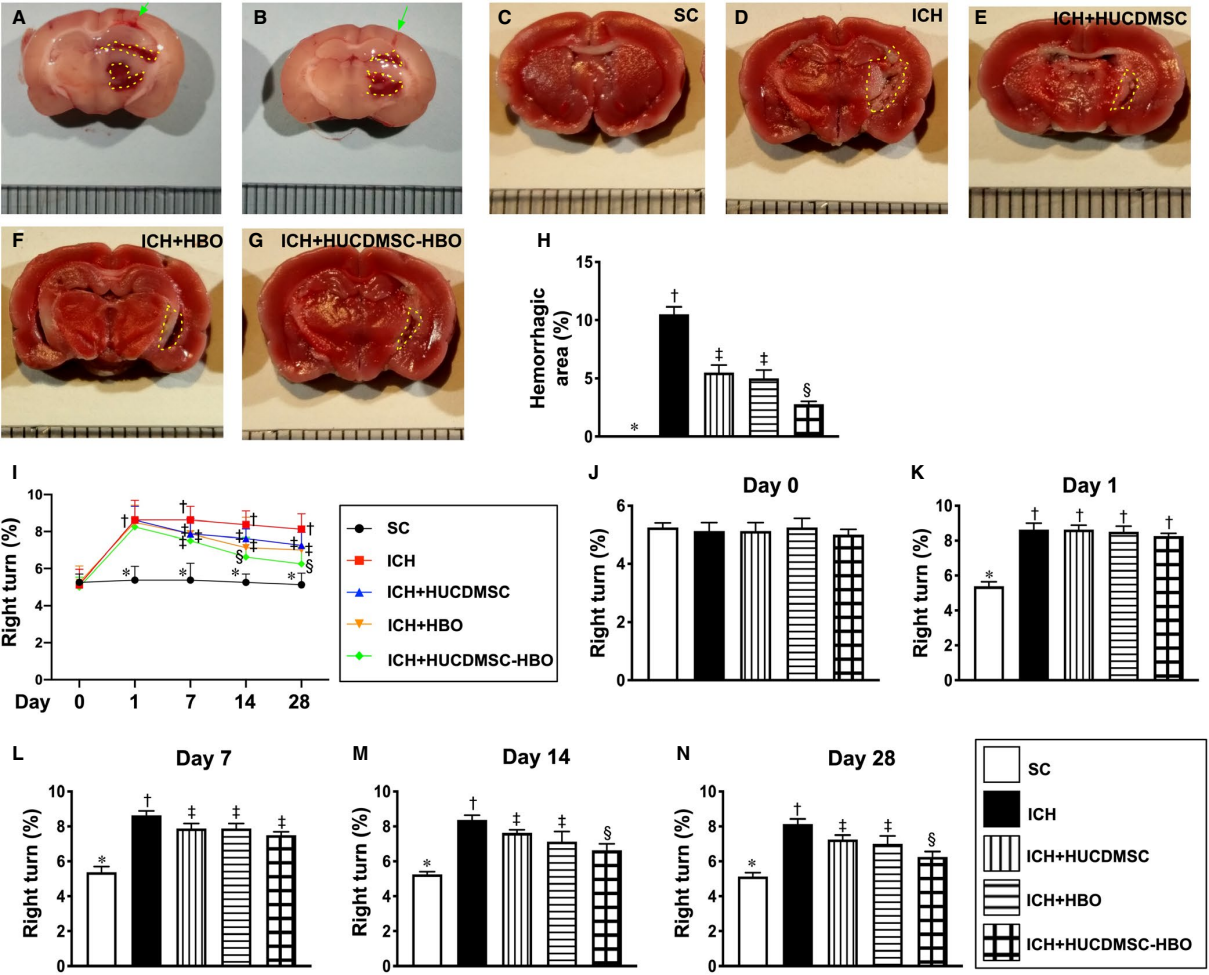
Intracerebral haemorrhagic area by day 14 after ICH induction and time courses of neurological function assessed by corner test. A and B, Illustrating two whole cross sections of brain which demonstrated intracranial injection of type IV collagenase successfully induced intracranial haemorrhage (red colour) (yellow dotted lines). Green arrow indicated the needle track. C‐G, Illustrating the triphenyltetrazolium chloride (TTC) (100×) of whole brain cross section for identification of percentage of intracerebral haemorrhagic area (the yellow dotted line indicated the boundary of the area) by day 14 after ICH induction (n = 4). H, Statistical analysis of summated (four brain cross sections in each animal) intracerebral haemorrhagic area, * vs. other groups with different symbols (†, ‡, §), *P* <.001. I, Illustrating the corner test for determining neurological function among days 0, 1, 7, 14 and 28 after acute ICH induction. J, Statistical analysis of neurological function (ie by corner test) by day 0, *P* >.5. K, Statistical analysis of neurological function day 1, * vs. †, *P* <.001. L, Statistical analysis by day 7, * vs. other groups with different symbols (†, ‡), *P* <.0001. M, Statistical analysis by day 14, * vs. other groups with different symbols (†, ‡, §), *P* <.001. N, Statistical analysis by day 28, * vs. other groups with different symbols (†, ‡, §), *P* <.0001. n = 6 for each group. All statistical analyses were performed by one‐way ANOVA, followed by Bonferroni multiple‐comparison post hoc test. Symbols (*, †, ‡, §) indicate significance (at 0.05 level). ICH, intracerebral haemorrhage; HUCDMSC, human umbilical cord‐derived mesenchymal stem cell; HBO, hyperbaric oxygen; SC, sham‐operated control

First, to assess the early stage of intracerebral haemorrhagic zone, the whole brain cross section was stained by TTC by day 14 after ICH induction (Figure 1A,B). The result demonstrated that the intracranial haemorrhagic area was lowest in group 1 (SC), highest in group 2 (ICH only) and significantly lower in group 5 (ICH +HUCDMSC + HBO) than in group 3 (ICH +HUCDMSC) and 4 (ICH +HBO), but it showed no difference between the groups 3 and 4 (Figure 1C‐H).

Next, to elucidate the therapeutic impact of HUCDMSC‐HBO on preservation of the neurological function (ie by corner test), the time courses of corner test (Figures 1I) were performed for the animals in each group. The results showed that by day 0 prior to ICH induction, the neurological function did not differ among the five groups (Figure 1J). However, by day 1, the neurological function was significantly impaired in groups 2 to 5 than in group 1, but it showed no difference among the groups 2 to 5 (Figure 1K). However, by days 7, 14 and 28, the neurological function was significantly impaired in group 2 than in group 1, while significantly progressively improved in groups 3 and 4 and further improved in group 5 (Figure 1L‐N).

### 3.2 Brain haemorrhagic volume and expression of NeuN+cells in brain haemorrhagic zone by day 28 after ICH induction

**FIGURE 2:**
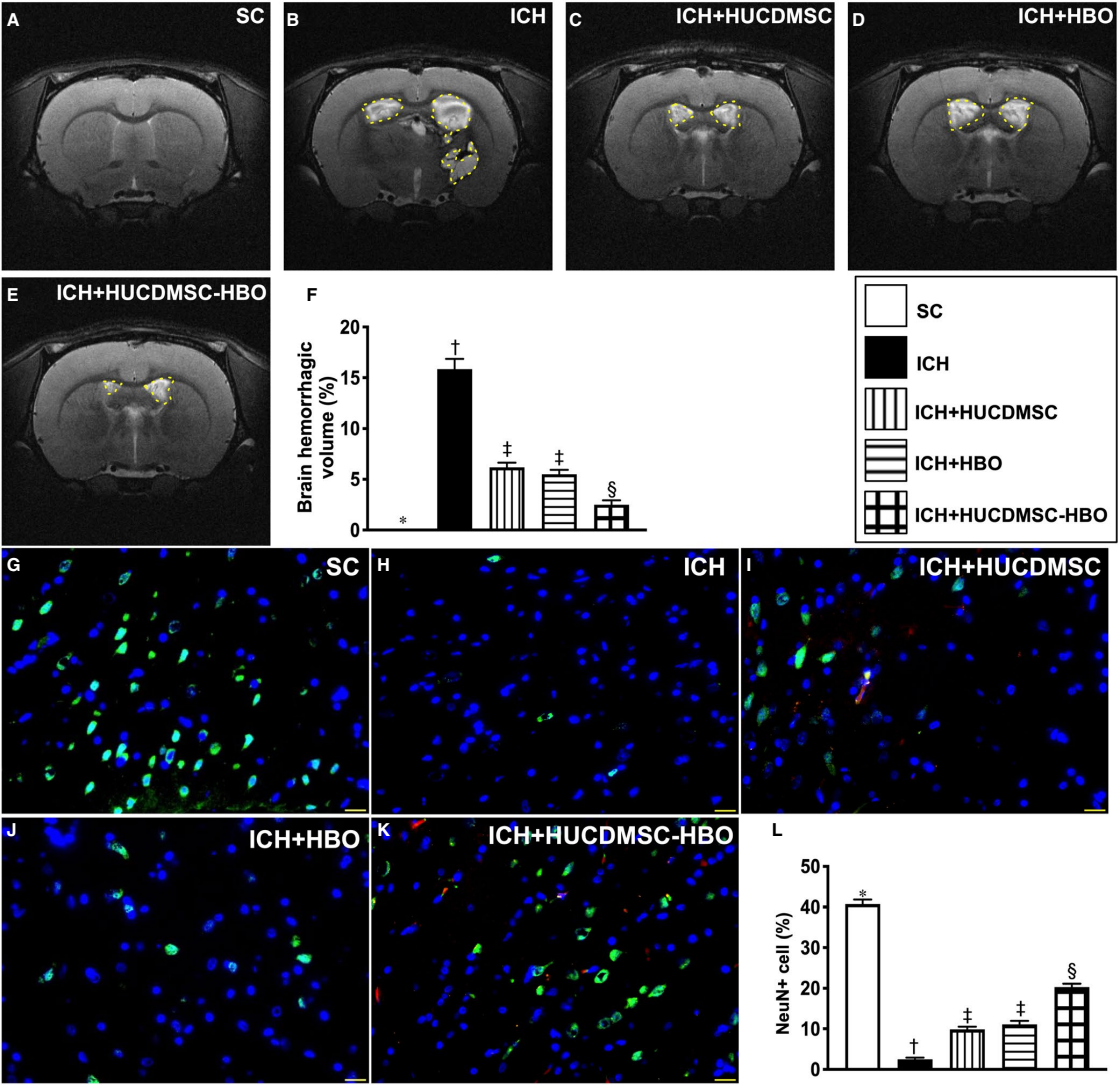
Brain haemorrhagic volume and expression of NeuN+cells in brain haemorrhagic zone by day 28 after ICH induction. A‐E, Illustrating the brain magnetic resonance imaging (MRI) finding for identification of brain haemorrhagic volume (white colour) (red dotted line area). F, Analytical result of brain haemorrhagic volume (ie the percentage of the whole brain volume), * vs. other groups with different symbols (†, ‡, §), *P* <.0001. G‐K, Illustrating the immunofluorescent microscopic finding (400x) for identification of expression of NeuN+cells (green colour). Red colour in (I) and (K) indicated the implanted cells. L, Analytical result of number of NeuN+cells, * vs. other groups with different symbols (†, ‡, §), *P* <.0001. Scale bar in right lower corner represents 20µm. All statistical analyses were performed by one‐way ANOVA, followed by Bonferroni multiple‐comparison post hoc test (n = 6 for each group). Symbols (*, †, ‡, §) indicate significance (at 0.05 level). ICH, intracerebral haemorrhage; HUCDMSC, human umbilical cord‐derived mesenchymal stem cell; HBO, hyperbaric oxygen; SC, sham‐operated control

To further evaluate the anatomical integrity of brain parenchyma, we utilized the brain MRI instrument. As we expected, by day 28 the brain MRI demonstrated that the BHV (Figure 2A‐E) was lowest in group 1, highest in group 2 and significantly lower in group 5 than in groups 3 and 4 (Figure 2F). However, this parameter did not differ between groups 3 and 4 (Figure 2F).

To realize the ultrastructure of the brain architecture in these animals, the high manifested IF microscopic analysis was done in the present study. The result revealed that number of NeuN+cells (Figure 2G‐K), an indicator of neurons, was highest in group 1, lowest in group 2 and significantly higher in group 5 than in groups 3 and 4 but it showed no difference between groups 3 and 4 (Figure 2L).

### 3.3 Impact of HUCDMSCs and HBO therapies on up‐regulating the angiogenesis capacity in brain parenchyma by day 28 after IHC induction

**FIGURE 3:**
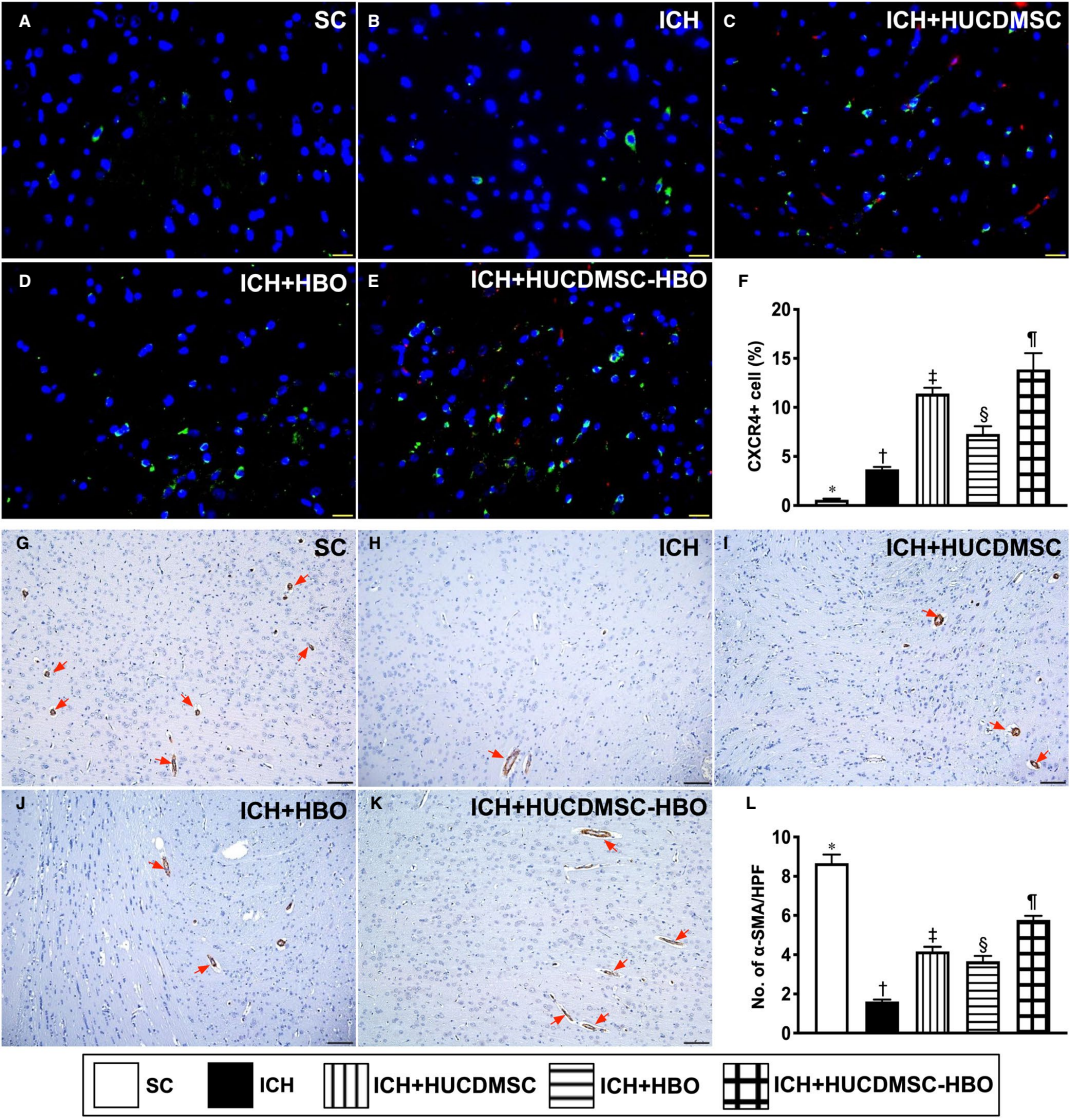
HUCDMSCs and HBO therapies enhanced angiogenesis in brain parenchyma by day 28 after ICH induction. A‐E, Showing the immunofluorescent microscopic finding (400×) for identification of cellular expression of CXCR4 (green colour). Red colour in (C) and (E) indicated the implanted cells. F, Analytical result of number of CXCR4+ cells, * vs. other groups with different symbols (†, ‡, §, ¶), *P* <.0001. Scale bar in right lower corner represent 20µm. G‐K, Showing the alpha smooth muscle actin (α‐SMA) stain (100×) for identifying the small vessels (grey colour) in brain parenchyma (red arrows). Analytical result of number of small vessels (ie diameter ≤ 25 μM), * vs. other groups with different symbols (†, ‡, §), *P* <.0001. Scale bar in right lower corner represent 20 µm. All statistical analyses were performed by one‐way ANOVA, followed by Bonferroni multiple‐comparison post hoc test (n = 6 for each group). Symbols (*, †, ‡, §, ¶) indicate significance (at 0.05 level). ICH, intracerebral haemorrhage; HUCDMSC, human umbilical cord‐derived mesenchymal stem cell; HBO, hyperbaric oxygen; SC, sham‐operated control

By using the IF microscopic examination, we further determined the angiogenesis ability of HUCDMSC‐HBO therapy in ischaemic zone. The result showed that the expression of CXCR4+ cells (Figure 3A‐E), an index of endothelial progenitor cells that play a crucial role for angiogenesis, was notably progressively increased from groups 1 to 5, implicating an intrinsic response to ischaemic stimulation that could be more augmented by HUCDMSC‐HBO therapy (Figure 3F). Additionally, the α‐SMA stain demonstrated that the number of small vessels [ie defined as the diameter ≤25 micrometre (μm)] (Figure 3G‐K), an indicator of angiogenesis/neovascularization, was highest in group 1, lowest in group 2 and significantly higher in group 5 than in groups 3 and 4 but it did not differ between groups 3 and 4 (Figure 3L).

**FIGURE 4:**
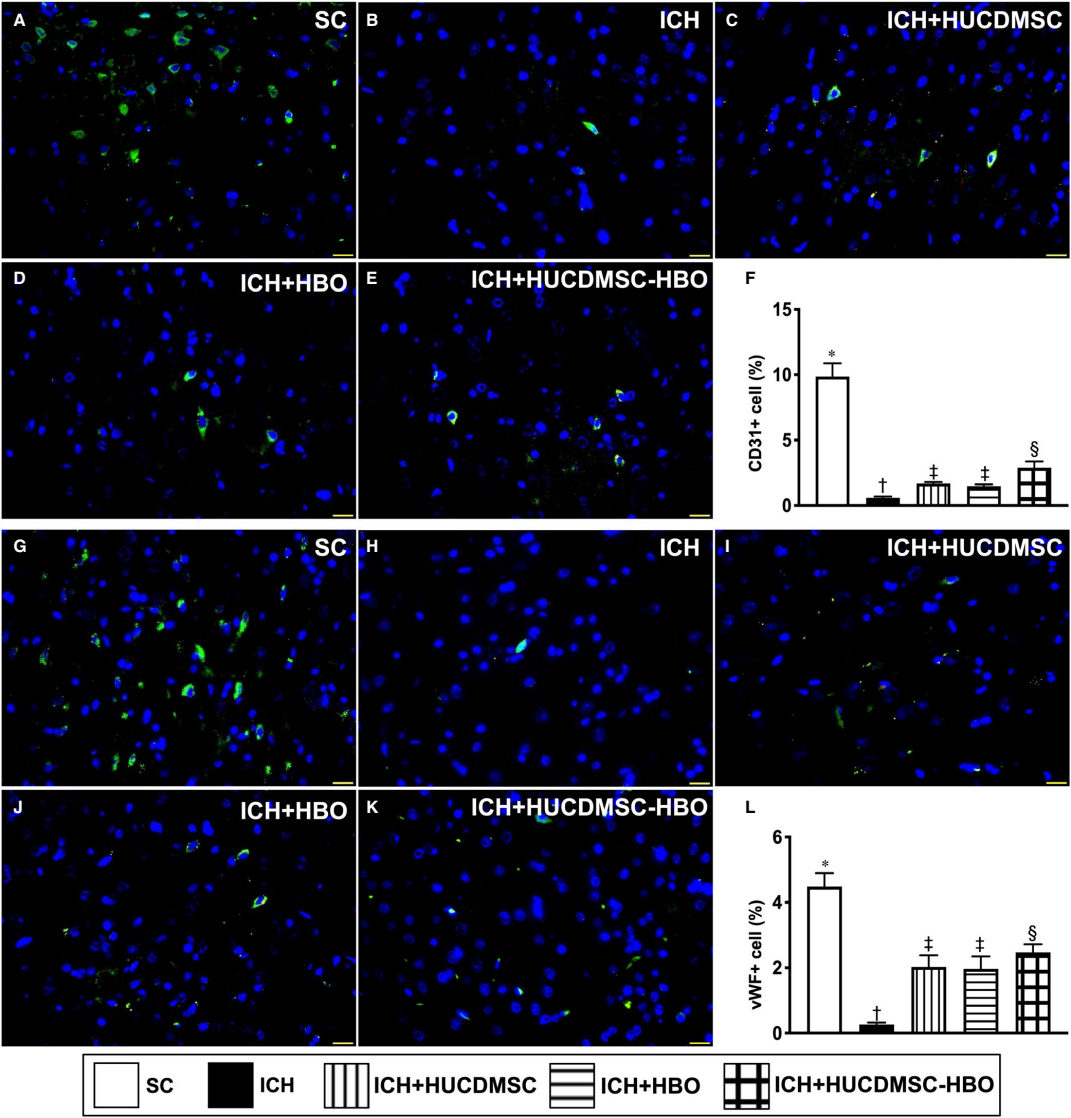
HUCDMSCs and HBO therapies enhanced the expressions of endothelial cell surface markers in brain parenchyma by day 28 after ICH induction. A‐E, Demonstrating the immunofluorescent (IF) microscopic finding (400×) for identification of CD31+ cells (green colour). F) Analytical result of number of CD31+ cells, * vs. other groups with different symbols (†, ‡, §), *P* <.0001. G‐K, Demonstrating the IF microscopic finding (400x) for identification of von Willebrand factor (vWF)+ cells (green colour). L, Analytical result of number of vWF+cells, * vs. other groups with different symbols (†, ‡, §), *P* <.0001. Scale bar in right lower corner represent 20µm. All statistical analyses were performed by one‐way ANOVA, followed by Bonferroni multiple‐comparison post hoc test (n = 6 for each group). Symbols (*, †, ‡, §) indicate significance (at 0.05 level). ICH, intracerebral haemorrhage; HUCDMSC, human umbilical cord‐derived mesenchymal stem cell; HBO, hyperbaric oxygen; SC, sham‐operated control

On the next stop, we also utilized the IF microscopic examination to identify the expressions of endothelial cell surface biomarkers of CD31 (Figure 4A‐E) and vWF (Figure 4G‐K), an integrity of endothelial cells. The result showed that these cellular expressions exhibited an identical pattern of number of small vessels among the five groups (Figure 4F,L).

### 3.4 Cellular and molecular level of inflammatory downstream signalling by day 28 after IHC induction

**FIGURE 5:**
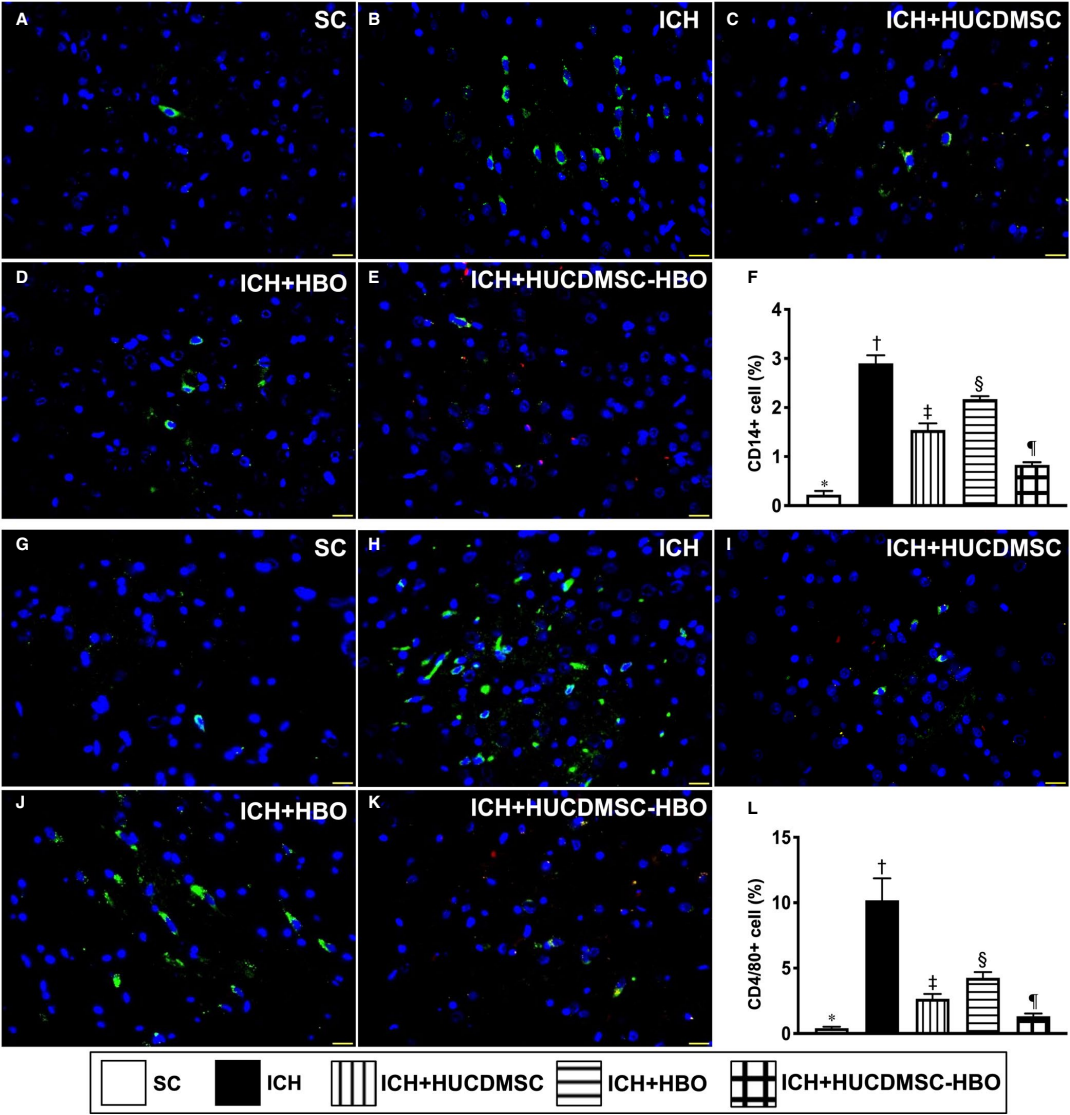
Inflammatory cell infiltration in brain parenchyma by day 28 after ICH induction. A‐E, Illustrating the immunofluorescent (IF) microscopic finding (400x) for cellular expression of CD14 (green colour). Red colour indicated the implanted cells. F, Analytical result of number of CD14+ cells, * vs. other groups with different symbols (†, ‡, §), *P* <.0001. G‐K, Illustrating the IF microscopic finding (400x) for cellular expression of F4/80 (green colour). L, Analytical result of number of CD4/80+ cells, * vs. other groups with different symbols (†, ‡, §), *P* <.0001. Scale bar in right lower corner represent 20 µm. All statistical analyses were performed by one‐way ANOVA, followed by Bonferroni multiple‐comparison post hoc test (n = 6 for each group). Symbols (*, †, ‡, §) indicate significance (at 0.05 level). SC =sham‐operated control; ICH, intracerebral haemorrhage; HUCDMSC, human umbilical cord‐derived mesenchymal stem cell; HBO, hyperbaric oxygen; SC, sham‐operated control

It is well recognized that tissue ischaemia and necrosis would always induce inflammatory reactions. Thus, we evaluated the protein and cellular level of inflammatory responses. As expected, the cellular expressions of CD14 (Figure 5A‐E) and F4/80 (Figure 5G‐K) in brain tissue, two indicators of inflammation, were highest in group 1, lowest in group 1, significantly lower in group 5 than in groups 3 and 4 and significantly lower in group 3 than in group 4.

**FIGURE 6:**
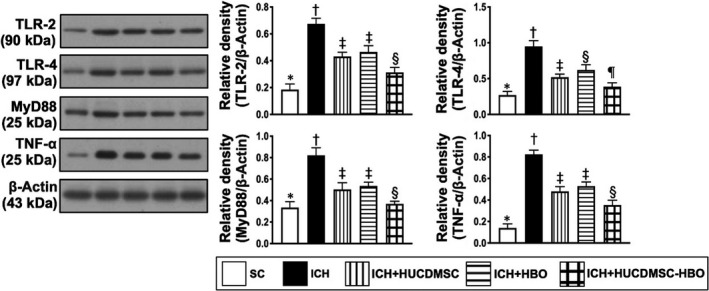
Protein expressions of inflammatory signalling in brain parenchyma by day 28 after ICH induction. Protein expression of Toll‐like receptor 2 (TLR‐2), TLR‐4, myeloid differentiation primary response 88 (MyD88) and tumour necrosis factor (TNF)‐α, * vs. other groups with different symbols (†, ‡, §), *P* <.0001. All statistical analyses were performed by one‐way ANOVA, followed by Bonferroni multiple‐comparison post hoc test (n = 6 for each group). Symbols (*, †, ‡, §, ¶) indicate significance (at 0.05 level). ICH, intracerebral haemorrhage; HUCDMSC, human umbilical cord‐derived mesenchymal stem cell; HBO, hyperbaric oxygen; SC, sham‐operated control

**FIGURE 7:**
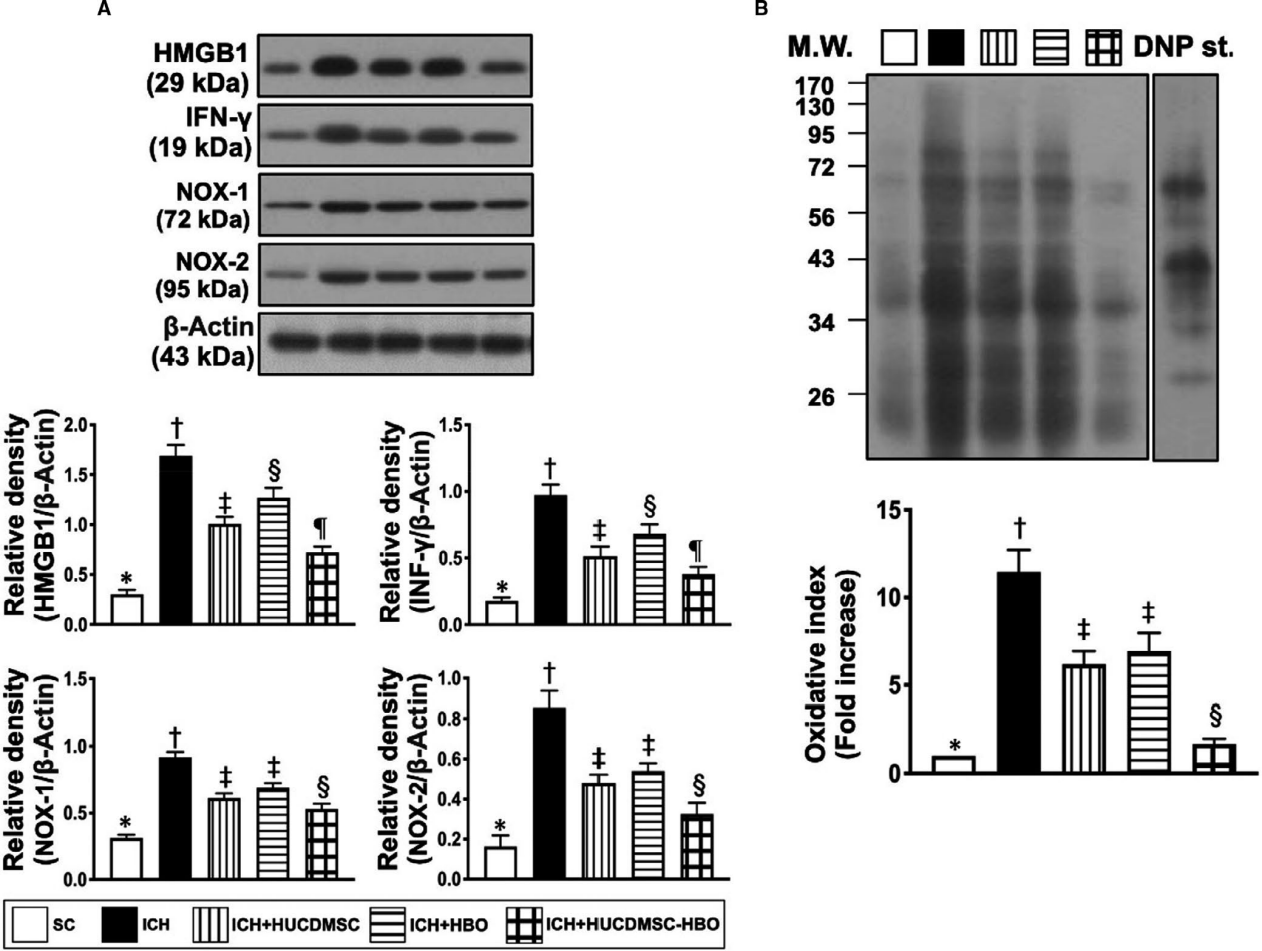
Protein expressions of inflammatory signalling and oxidative stress markers in brain parenchyma by day 28 after ICH induction. A, Protein expression of high mobility group box 1 (HMGB1), interferon (INF)‐γ, NOX‐1 and NOX‐2, * vs. other groups with different symbols (†, ‡, §), *P* <.001. B, The oxidized protein expression, * vs. other groups with different symbols (†, ‡, §), *P* <.0001 (Note: the left and right lanes shown on the upper panel represent protein molecular weight marker and control oxidized molecular protein standard, respectively). DNP, 1‐3 dinitrophenylhydrazone; M.W, molecular weight. All statistical analyses were performed by one‐way ANOVA, followed by Bonferroni multiple‐comparison post hoc test (n = 6 for each group). Symbols (*, †, ‡, §, ¶) indicate significance (at 0.05 level). ICH, intracerebral haemorrhage; HUCDMSC, human umbilical cord‐derived mesenchymal stem cell; HBO, hyperbaric oxygen; SC, sham‐operated control

**FIGURE 8:**
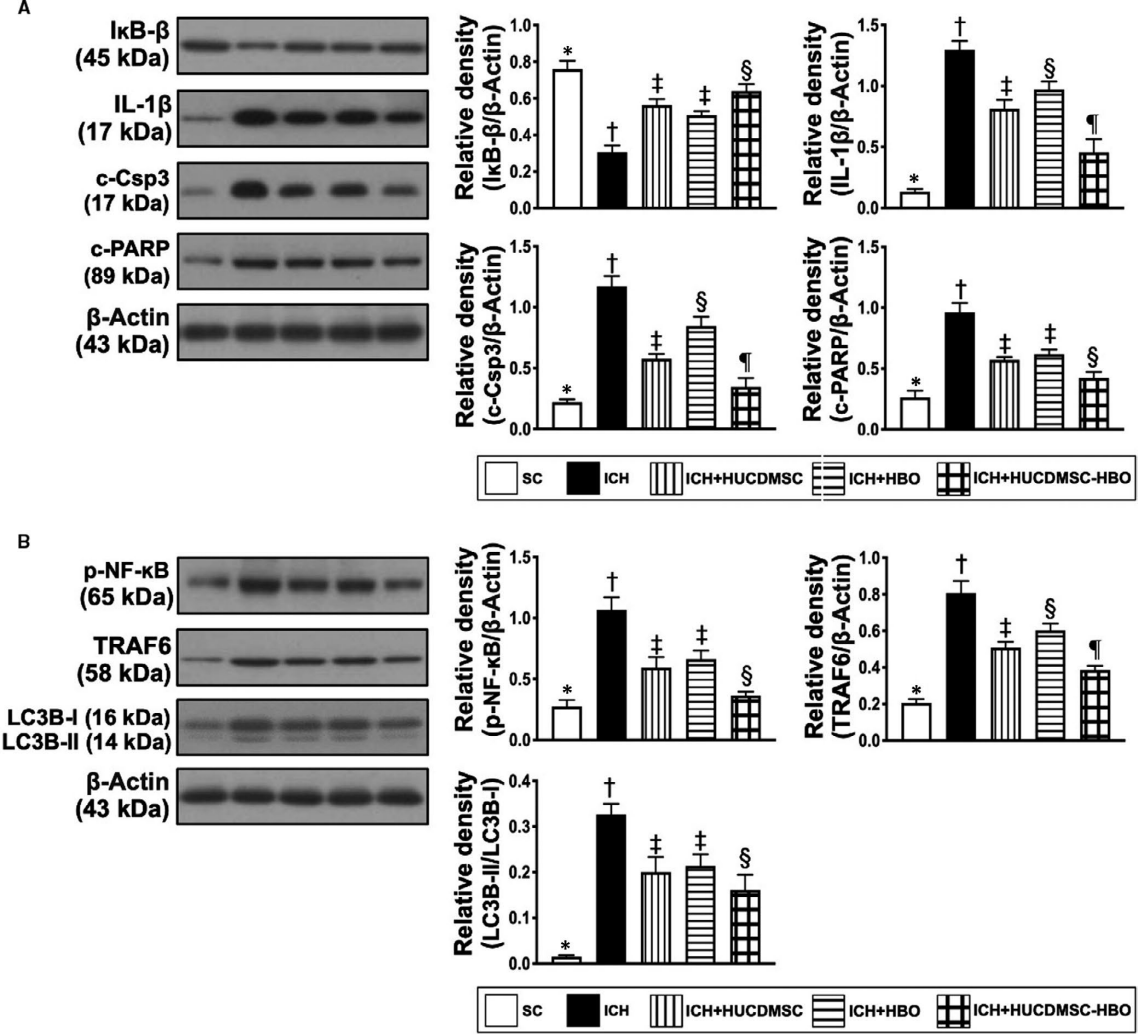
Protein level of oxidative stress and autophagic biomarkers by day 28 after ICH induction. A, Protein expressions of nuclear factor of kappa light polypeptide gene enhancer in B‐cell inhibitor beta (IκB‐ß), interleukin (IL)‐1ß, cleaved caspase 3 (c‐Cap3) and cleaved (c)‐PARP, * vs. other groups with different symbols (†, ‡, §), *P* <.001. B, Protein expression of phosphorylated (p) nuclear factor (p‐NF)‐κB, TNF receptor‐associated factor 6 (TRAF6) and ratio of LC3B‐II to LC3B‐I, * vs. other groups with different symbols (†, ‡, §), *P* <.0001. All statistical analyses were performed by one‐way ANOVA, followed by Bonferroni multiple‐comparison post hoc test (n = 6 for each group). Symbols (*, †, ‡, §, ¶) indicate significance (at 0.05 level). ICH, intracerebral haemorrhage; HUCDMSC, human umbilical cord‐derived mesenchymal stem cell; HBO, hyperbaric oxygen; SC, sham‐operated control

We then returned to see the molecular level of inflammation. Consistently, the Western blot analysis showed that the protein expressions of TLR‐2, TLR‐4, MyD88, HMGB1 and TRAF6 (Figure 6, 7A and 8B), five indices of upstream inflammatory signalling, displayed a similar pattern of CD14+ cells among the five groups. Additionally, the protein expressions of TNF‐α, IFN‐γ, IL‐1ß, p‐NF‐κB and (Figure 6, 7A and 8A,B), four indicators of downstream inflammatory signalling, also exhibited a similar pattern of CD14+ cell, whereas the protein expression of IκB‐ß (Figure 8A), another indicator of downstream signalling, displayed an opposite pattern of CD14+ cells among the five groups.

### 3.5 Protein level of oxidative stress and autophagic biomarkers by day 28 after IHC induction

It is also well recognized that tissue ischemia and necrosis always elicit oxidative stress which, in turn, induces apoptosis and up‐regulated the autophagy. Based on this concept, we performed Western blot analysis. The result demonstrated that the protein expressions of NOX‐1, NOX‐2 and oxidized protein (Figure 7A), three indices of oxidative stress, were lowest in group 1, highest in group 2 and significantly lower in group 5 than in groups 3 and 4, but they showed no difference between groups 3 and 4. Additionally, the protein expressions of cleaved caspase 3 (Figure 8A), and indicator of apoptosis, was lowest in group 1, highest in group 2, significantly lower in group 5 than in groups 3 and 4 and significantly lower in group 3 than in group 4. On the other hand, the cleaved PARP (Figure 8A), another indicator of apoptosis and the ratio of LC3B‐II/LC3B‐I (Figure 8B), an indicator of autophagic biomarker, exhibited an identical pattern of oxidative stress among the five groups.

## 4 DISCUSSION

This study which investigated the therapeutic impact of HUCDMSC‐HBO on ICH rodent yielded several preclinical implications. First, HUCDMSC or HBO therapy substantially reduced the brain haemorrhagic area (ie estimated by TCC stain at early stage of ICH) and brain haemorrhagic volume (ie estimated by brain MRI at day 28 after ICH) and remarkably preserved neurological function (ie estimated by corner test). Second, the extensive woks were done in the present study and found that inflammatory signalling and oxidative stress could be the mainly underlying mechanism for brain damage after ICH induction. Third, therapeutic impact of HBO was comparable with HUCDMSC for protecting the brain architecture against haemorrhagic damage. Finally, the combined HUCDMSC‐HBO therapy was superior to either one alone on protecting the brain volume and neurological function in setting of ICH.

The effective treatment of severe ICH is still lacking. This could explain for why this disease entity is still a high cause of death and disability in adults worldwide.^1^, ^2^, ^3^ Interestingly, our recent studies^27^, ^28^ have shown that exogenous MSC treatment for rodent IS or brain haemorrhage was safe and without immune rejection. Of importance was that this MSC therapy effectively protected the brain from ischaemic‐related/haemorrhagic‐related injury.^27^, ^28^ Additionally, our other recent studies have shown that HBO therapy effectively preserved the brain infarct volume and neurological function in rat after IS.^30^, ^34^ The most important finding in the present study was that as compared with ICH group, the brain haemorrhagic volume and brain haemorrhagic zone were significantly preserved in IHC animals treated by HUCDMSCs and HBO. Of distinctive importance was that combined HUCDMSCs and HBO offer additional benefit than single one therapy alone on preserving the brain architecture and neurological integrity after ICH attack. Our findings, in addition to extending the findings of previous studies,^27^, ^28^, ^30^, ^34^ could, at least in part, explain for why the neurological function of ICH animals was better improved by combined HUCDMSC and HBO therapy than in either monotherapy.

**FIGURE 9:**
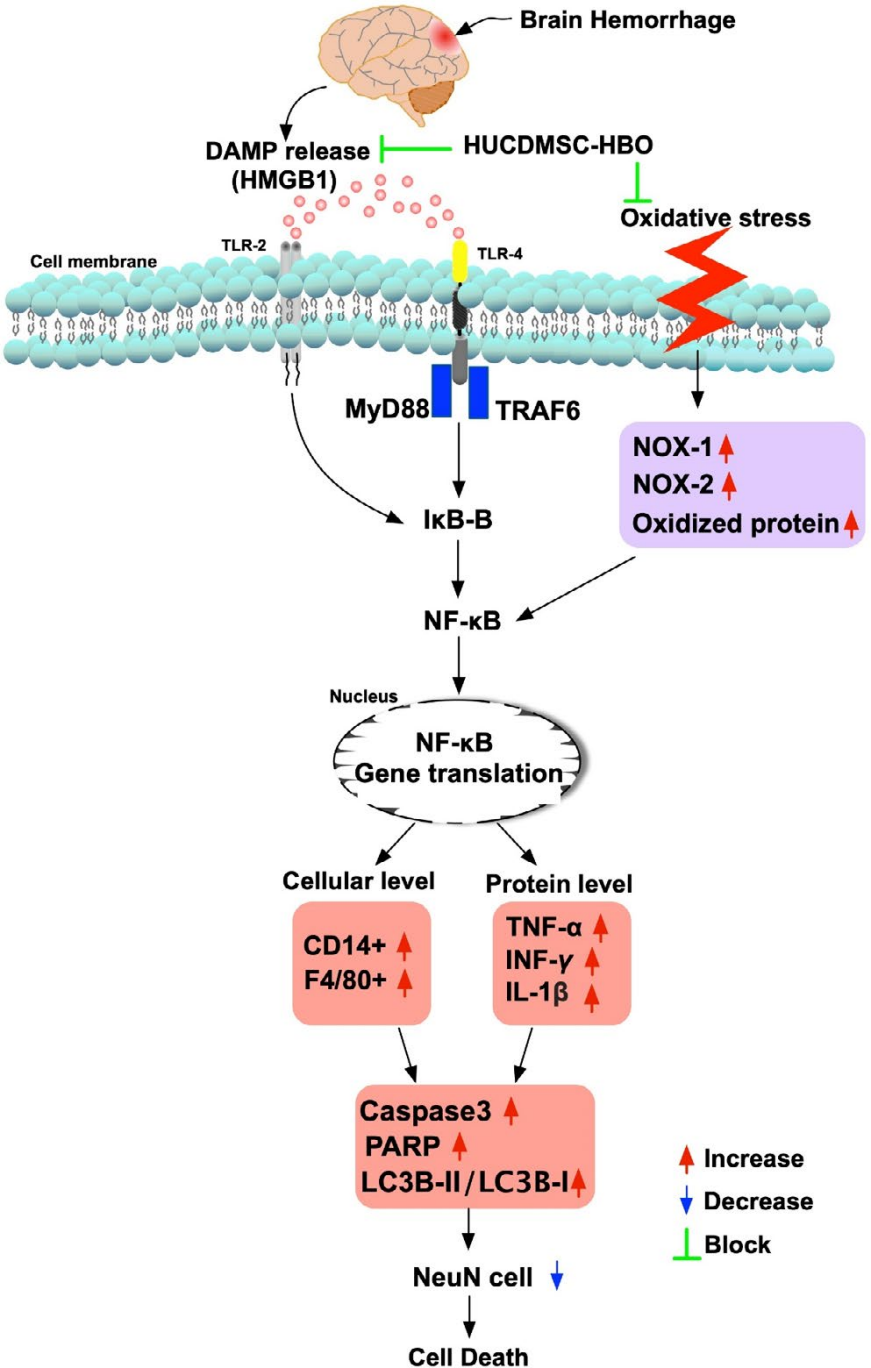
The proposed mechanism for how the intracerebral haemorrhage induced inflammatory signalling and oxidative stress and the impact of HUCDMSC +HBO therapy on improving the outcomes after IHC in rodent. DAMP, damage‐associated molecular patterns; HMGB1, protein expression of high mobility group box 1; TRAF6, TNF receptor‐associated factor 6; TLR, Toll‐like receptor

It is well documented that the mechanical damage is always immediately elicited after ICH which commonly wrecks the brain tissue as it enlarges.^2^ This is frequently associated with the pressure created enough by blood and surrounding brain oedema, resulting in a life‐threatening situation.^4^ The secondary damage is caused by cytotoxicity of blood,^5^, ^6^ impaired calcium homeostasis,^7^ excitotoxicity from excitatory neurotransmitters/glial cells^8^, ^9^ and up‐regulation of oxidative stress and inflammation.^6^, ^9^, ^10^, ^11^, ^12^, ^13^, ^14^ Additionally, an association between the up‐regulation of inflammation and oxidative stress and organ damage in setting of ischaemia or ischaemia‐reperfusion injury have been extensively investigated by previous studies.^19^, ^20^, ^21^, ^22^, ^23^, ^24^ An essential finding in the present study was that as compared with SC group, the brain haemorrhagic volume (ie caused mechanical damage) and the inflammatory downstream signalling and oxidative stress (ie caused by cellular and molecular perturbations) were remarkably up‐regulated in ICH animals (refer to Figure 9). Our findings, in addition to being consistent with the findings of the previous studies,^2^, ^19^, ^20^, ^21^, ^22^, ^23^, ^24^ could explain for why much higher apoptosis autophagic phenomenon and the unfavourable outcomes developed in the IHC animals and suggest that inflammatory signalling/oxidative stress plaid an ultimately crucial role on damage (ie second damage) of the brain architecture and neuron integrity after ICH.

The mechanisms for why MSCs and HBO therapy effectively protect the cells/tissues and organs from any ischaemia‐related damage always elicit high interest from scientists.^35^, ^36^ Currently, abundant data have shown that anti‐inflammation, immunomodulation and tissue regeneration as well as cytokine/paracrine effects could be the main mechanisms for MSCs therapy on safeguarding the cells/tissues and organs far away from inflammatory and ischaemic related damage.^27^, ^28^, ^36^ Our findings demonstrated even xenogenic HUCDMSCs (ie from human‐derived MSC to rodent) were still safe without any immune reaction and offered additional benefit to IHC rats (refer to Figure 9). In this way, our findings corroborated with the findings of previous studies.^27^, ^28^, ^36^ On the other hand, the mechanistic basis of HBO therapy on protecting the ischaemic cells/tissues and organs from ischaemic damage has been emphasized mainly as a result of up‐regulation of circulating levels of endothelial progenitor cells/MSCs, an increase in vascular permeability, high content of oxygen in ischaemic tissues and antiapoptotic and anti‐inflammatory effects.^27^, ^28^, ^36^ These findings ^27^, ^28^, ^36^ could explain for why a synergic effect of combined HUCDMSC +HBO therapy on reducing the brain haemorrhagic volume and improving the neurological function to be identified in the present study.

We proposed the therapeutic regimen of the multiple dosages of HUCDMSCs (ie like the regimen of antibiotics to be utilized for treatment the bacterial‐induced sepsis in our daily clinical practice) could be the key to success for remarkable preservation of the brain architecture and neurological function. Interestingly, our previous^36^ and recent^37^ studies have shown that early and multiple dosages of stem cell administration was the crucial role on reducing the post‐heart transplant acute rejection^36^ and preservation of the neurological function after acute ischaemic stroke ^37^ in rodent. In this way, the results of the present study, in addition to being comparable with the findings of our previous^36^ and recent^37^ studies, highlight that this regimen could be seriously considered in our future stem cell therapy for the patients.

### 4.1 Study limitations

This study has limitations. Despite a lot of works that had been done in the present study, the exact mechanisms of combined HUCDMSCs and HBO on preserving the brain architecture and neurological function were still not fully investigated. Based on the results of the present study, Figure 9 illustrated the proposed mechanism for how the HUCDMSC +HBO therapy on improving the outcomes after IHC in rodent. Second, although the short‐term outcomes (ie at a study period of 28 days) were attractive and promising, the long‐term impact of such kind of therapy remains uncertain.

## 5 CONCLUSIONS

The results of the present study demonstrated that combined HUCDMSC and HBO therapy offered a synergic effect on protecting the macro‐ and micro‐ultrastructure of the brain and preserving neurological integrity in rat after ICH.

## CONFLICTS OF INTEREST

The authors declare that they have no conflicts of interest.

## AUTHOR CONTRIBUTION

**Hon‐Kan Yip:** Investigation (equal); Methodology (equal); Resources (lead). **Kun‐Chen Lin:** Investigation (lead); Methodology (equal). **Pei‐Hsun Sung:** Methodology (equal). **John Y. Chiang:** Writing‐review & editing (equal). **Tsung‐Cheng Yin:** Methodology (equal). **Re‐Wen Wu:** Methodology (equal). **Kuan‐Hung Chen:** Resources (equal); Writing‐original draft (equal).

## Data Availability

The datasets of present study can be available from the corresponding author upon request.
